# Two cleft palate simulators of Furlow double-opposing Z- palatoplasty: a comparative study

**DOI:** 10.1186/s12893-023-02201-5

**Published:** 2023-10-04

**Authors:** Sadam Ahmed Elayah, Mohammed Qasem Al-Watary, Karim Ahmed Sakran, Yang Chao, Li Jingtao, Huang Hanyao, Yang Li, Bing Shi

**Affiliations:** 1 State Key Laboratory of Oral Diseases & National Center for Stomatology & National Clinical Research Center for Oral Diseases, West China Hospital of Stomatology, Sichuan University, Chengdu 610041, China; 2 Department of Oral and Maxillofacial Surgery, Cleft Lip and Palate Center, Faculty of Dentistry, Jiblah University for Medical and Health Sciences, Ibb, Yemen

**Keywords:** Cleft palate, Surgical procedures, Training, Simulation, Residency

## Abstract

**Purpose:**

This study aimed to evaluate the efficiency of the porcine tongue for palatoplasty simulation compared to 3D-printed simulators and their surgical education role.

**Materials and methods:**

A total of 18 senior cleft surgeons participated in a palatoplasty simulation-based workshop conducted using porcine tongue simulators and 3D-printed simulators. This workshop consisted of a didactic session followed by a hands-on simulation session. Each participant independently used both simulators to perform Furlow double-opposing Z-plasty, which was assessed and scored by senior cleft surgeons using a scoring system including organizational flexibility and ductility, anatomical design simulation, proper incision, proper suturing, and convenience of operation. A paired *t* test was used for data statistical analysis and a P value < 0.05 was regarded as a statistically significant difference.

**Results:**

All senior cleft surgeons strongly agreed that the simulation-based workshop was a valuable learning experience, and both simulators were useful and easy to manipulate (P = 1.00). The results of this comparative study showed that a porcine tongue palatoplasty simulator had an effectively significant difference in terms of organizational flexibility and ductility (P = 0.04), and suturing was better than the 3D-printed palatoplasty simulator (P < 0.01). There were no significant differences between the simulators regarding anatomical design simulation (P = 0.76) and incision simulation (P = 0.65).

**Conclusion:**

Both porcine tongue simulator and 3D-printed simulator have their unique strengths in surgical education for palatoplasty. Thus, the combined use of a porcine tongue and a 3D-printed cleft palate simulators are efficient as an educational model to practice Furlow double-opposing Z- palatoplasty. The porcine tongue simulators are superior in terms of organizational flexibility, ductility, and suturing simulators, while with the 3D-printed simulator, various palatoplasty techniques can be repeatedly practiced with better-simulated face and oral cavity.

## Introduction

The widespread attention on patient safety and resident mental health has raised the demand for educational materials outside the operating room. A simulation is a helpful tool for evaluating and enhancing surgical skills in a safe and controlled environment. It was originally used in general surgery [1], and it has been observed to provide better advances in skills and knowledge when compared to traditional teaching methods such as self-directed reading and the usage of digital images [2]. As such, multiple cleft palate simulators with realistic tactile characteristics and precise anatomical simulation have proven useful in teaching cleft palate repair techniques to enhance the understanding and confidence level of trainees as they progress through their residency program [3–5].

Simulation-based training (SBT) has evolved as an essential component of postgraduate surgical education. It is effective in teaching procedural skills in a safe, no-risk training environment and therefore has been incorporated into multiple residency curricula [1]. Not surprisingly, using the SBT approach in cleft lip and palate surgery training programs will enhance the trainee’s skills in performing different techniques with minimum morbidities, such as fistula formation, poor scarring, and velopharyngeal insufficiency [6, 7]. Cleft palate simulators are highly accepted and have become a crucial methodology for developing and polishing the skills and competencies of surgeons at all stages of their careers [8]. In terms of construction, simulators can be constructed using synthetic, animal-derived, cadaver-derived, or virtual models, each with its advantages and disadvantages [1, 9]. One example is porcine tongue simulators [10–12], they offer an exceptional platform for realistic tissue simulation, closely resembling human tissue in terms of texture, elasticity, and consistency. This similarity makes them invaluable for honing surgical skills, particularly for procedures like palatoplasty that involve delicate manipulation of soft tissues. Moreover, their accessibility and cost-effectiveness make porcine tongue simulators a pragmatic choice for surgical education programs operating within budget constraints. Importantly, they offer a solution to ethical concerns surrounding the use of human cadavers in training, thus gaining wider acceptance in medical institutions.

While animal-derived simulators for palatoplasty surgical simulation are limited [10, 13, 14], synthetic simulators in the form of various types of 3D-printed models are available for teaching and learning palatoplasty [3, 7, 15, 16]. They can faithfully replicate the face and oral cavity and their customizability is allowing educators to design and create specific surgical scenarios tailored to the needs of trainees. Importantly, the use of 3D-printed simulators enhances safety in medical training.

Recently, authors described a porcine tongue for surgical simulation of double-opposing z-plasty [13]. In addition, authors developed a 3D-printed model for the surgical simulation of palatoplasty. An ideal simulation model should be able to be easily manufactured, reasonably priced, and accurately represent the anatomy. Currently available cleft surgery simulators are either too simple or too costly to be of use for surgical training [9]. Thus, this study aimed to evaluate the efficiency of the porcine tongue for palatoplasty simulation compared to 3D-printed simulators and their surgical education role.

## Materials and methods

### Trainees

This study was ethically approved from the institutional ethics committee at the Advanced Science Research Center, Department of Animal Resources, Sichuan University (No. WCHSIRB-D-2022-409).

Based on Helsinki guidelines, a total of 18 senior cleft surgeons participated in a palatoplasty simulation-based workshop conducted on the porcine tongue simulator and the 3D-printed simulator. The primary objective of this study is to compare the efficiency of two cleft palate simulators used in a palatoplasty simulation-based workshop. To achieve this objective, authors deliberately chose to involve senior cleft surgeons with substantial experience in performing palatoplasty procedures. Senior cleft surgeons have a deep understanding of the clinical implications of surgical techniques and can better evaluate whether the simulators would be useful in educating and training future generations of cleft surgeons.

The simulation workshop consisted of a didactic session given by an expert surgeon on cleft palate anatomy and surgical repair techniques repair (Bing Shi), followed by a hands-on simulation session, and has been written informed consents were obtained from all trainees and instructors. Each participant independently used both simulators to perform Furlow double-opposing Z-plasty, which results in acceptable velopharyngeal function, making it one of the most frequently utilized primary palatoplasty techniques [17]. The key surgical steps that may be conducted using simulators are as follows: (A) outlining the incisional design, (B) separation of the simulated oral and nasal layers, (C) elevation of oral Z-flaps and design of the nasal Z-flaps incision, (C) suturing of the nasal layer, including Z-flaps, and (D) closure of the oral layer, including Z-flaps.

All experimental methods were carried out at the Advanced Science Research Center, Department of Animal Resources, with approval from Animal Research Committee, Sichuan University in accordance with the ARRIVE guidelines.

### Cleft palate simulators

Of a total of 36 simulators, 18 were dead porcine tongue simulators bought from a supermarket, while the other 18 were 3D-printed simulators for Furlow palatoplasty. Porcine tongue simulators provide a highly realistic tissue simulation due to their similarity to human tissue in terms of texture, elasticity, and consistency. This makes them an excellent choice for practicing palatoplasty procedures, which involve delicate manipulation of soft tissues [10].

**Fig. 1:**
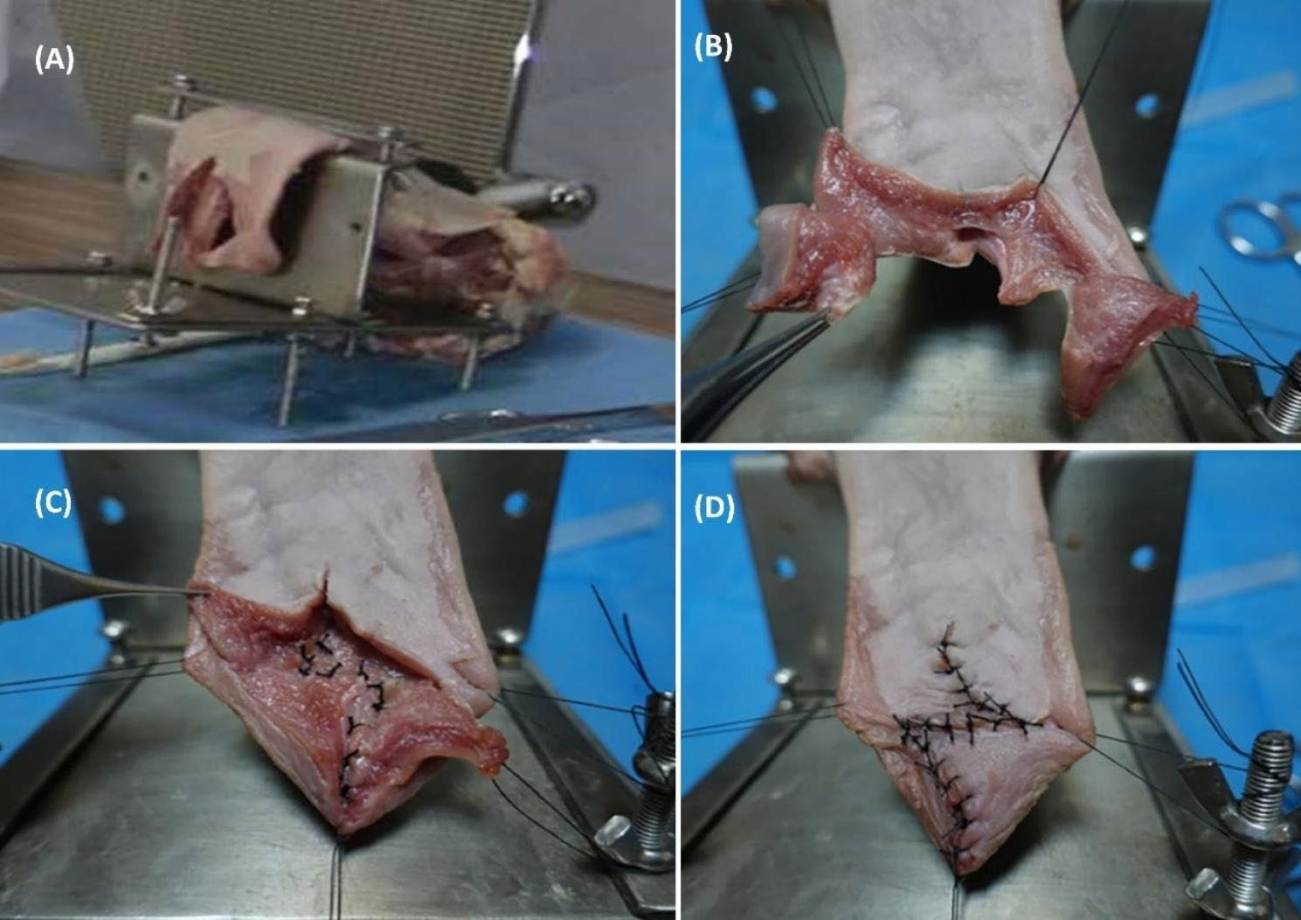
The surgical procedure of double-opposing Z-plasty on porcine tongue; **(A)**The porcine tongue fixated on the holder with being cut in the middle part. **(B)** To simulate the Z-plasty flaps, Preparation of two myomucosal flaps and two mucosal flaps. **(C)** Suturing of Nasal Z-plasty flap. **(D)** Suturing of Oral Z-plasty flap

**Fig. 2:**
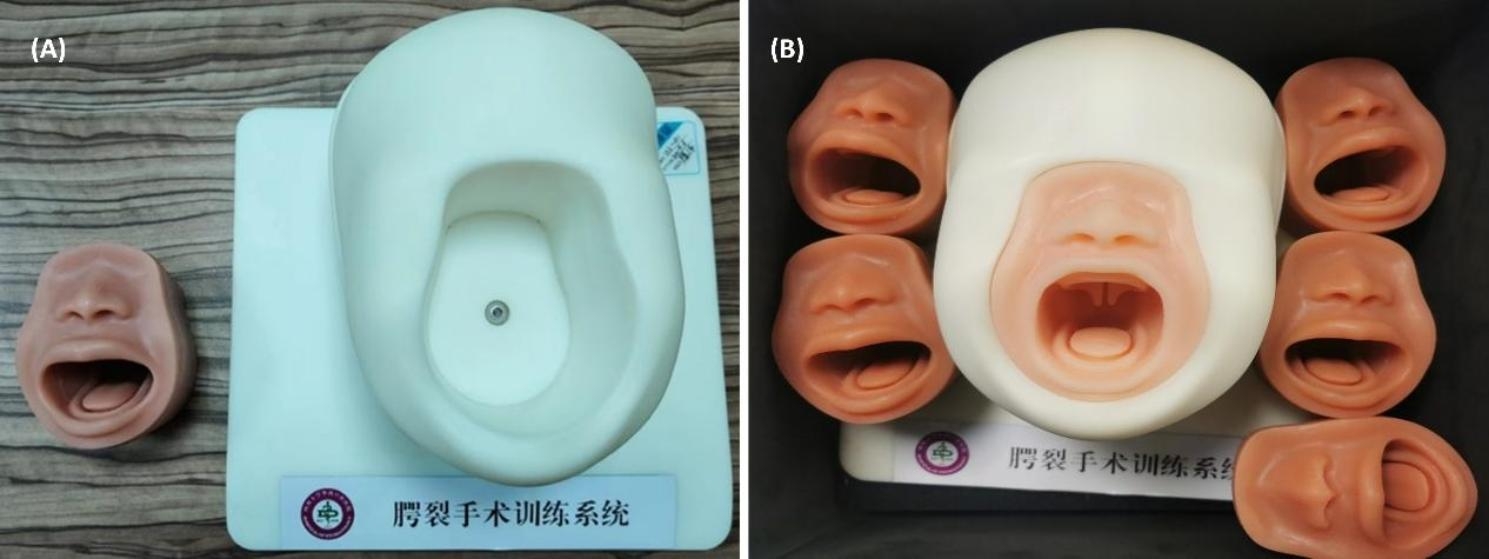
3D printed cleft palate simulator; **(A)** Parts of 3D printed cleft palate simulator. **(B)** 3D printed cleft palate simulator with the mold simulating the infant’s head

A porcine tongue was formed the first group of this study and prepared to simulate the cleft palate using the following steps [18]: a 5 cm long piece was taken from the tip of a porcine tongue and then fixed in 4% formaldehyde (PFA). Coronal and sagittal sections were created and then dehydrated and embedded. Five-micron-thick paraffin sections were cut and stained with Masson staining. The free part of the tip of the tongue was cut approximately 3–4 cm along the midline to simulate the soft palate fissure. (Fig. 1). The second group was formed by synthetic simulators in the form of 3D-printed simulators, a standard infant head mold with a replaceable 3D-printed cleft palate model (Fig. 2). 3D-Printed Simulators mimic the face and oral cavity, which is crucial for surgical training. Surgeons can gain a better understanding of the spatial relationships and structures they will encounter during palatoplasty [19].

### Assessment of cleft palate repair simulation

The simulated Furlow palatoplasty was assessed and scored by senior cleft surgeons using a scoring system, including organizational flexibility and ductility, anatomical design simulation, proper incision, proper suturing, and convenience of operation.

The scoring system ranged from 0 to 10 points, evaluated according to predefined objective criteria, as shown in (Table 1). A value close to 10 means that the simulator is close to the actual palatoplasty. In addition, at the end of the simulation, each senior cleft surgeon completed a workshop evaluation questionnaire including which one of the palatoplasty simulators was preferred and why (Table 2).

**Table 1:** Evaluation criteria for the Furlow palatoplasty simulation with the results of the participants responses regarding the comparison of the two simulators

Evaluation criteria	Porcine tongue Simulator	3D-Printed Simulator	*P-* Value
	Mean ± SD	Mean ± SD	
Organizational flexibility and ductility	8.61 ± 1.17	7.35 ± 1.46	**0.04**
Anatomical design simulation	8.33 ± 1.22	7.92 ± 1.50	0.76
Incision simulation	8.22 ± 1.48	7.50 ± 1.51	0.65
Suturing simulation	8.94 ± 0.80	6.92 ± 1.51	**<0.01**
Convenience of operation	8.28 ± 0.91	8.17 ± 1.80	1.00

**SD:** Standard deviation

**Table 2:** The results of the questionnaire surveys of surgeons participated in the simulation-based workshop regarding the comparison of the two simulators

Evaluation Form Item	Strongly Agree (n 18), (100%)	Agree (n 18), (100%)
The simulation workshop was a valuable learning experience	(18), (100%)	
The cleft palate repair simulation based-learning workshop was a useful exercise	(18), (100%)	
The cleft palate simulator should be integrated into senior residency or fellowship training	(18), (100%)	
I would use the cleft palate simulator again for practice	(15), (83.3%)	(3), (16.6%)
I would recommend this simulation workshop to my colleagues	(18), (100%)	
**What is your favorite simulator?**		
Porcine tongue simulator	8 (44%)	
3D-Printed simulator	8 (44%)	
Both simulators	2(11%)	

**n**: number, **3D**: 3-dimensional

### Data analysis

SPSS v. 25 statistical software [20, 21] was used to conduct the statistical analysis of the results of the current study. A paired *t* test was used for data statistics, and a P value < 0.05 was regarded as a statistically significant difference.

## Results

A total of 18 senior cleft surgeons completed the simulation-based workshop with completed evaluated questionnaires. All senior cleft surgeons strongly agreed that the simulation-based workshop was a valuable learning experience and that both simulators were useful and easy to practice (P = 1.00) (Table 2). The objective evaluation results of this comparative study (Table 1) showed that a porcine tongue palatoplasty simulator had an effectively significant difference in terms of organizational flexibility and ductility (P = 0.04), and suturing (P < 0.01). There were no significant differences between the simulators in terms of anatomical design simulation (P = 0.76) and incision simulation (P = 0.65). Out of the 18 participating surgeons, eight (44.4%) preferred the porcine tongue simulator because of its lower price and better operability, while another eight surgeons preferred the 3D-printed simulator because of its good face and oral cavity simulation and operability, and the two remaining surgeons (11.1%) preferred both simulators. Overall, 17 surgeons (94.4%) reported that if the 3D-printed simulator is well improved to simulate the palatal muscles and tissues, it will be chosen and preferred compared to the porcine tongue simulator.

## Discussion

While cleft palate is one of the most common congenital defects, surgical correction continues to remain challenging and may result in serious complications when performed by inexperienced surgeons [18, 19]. By repetition and anatomical replication in a controlled environment, simulation allows residents to enhance both their cognitive and physical skills. It also alleviates many of the restrictions that exist in the operating room, such as time, the teaching strategy of the attending surgeon, and trainee learning style [22–24].

The current study showed that palatoplasty conducted on a porcine tongue simulator was superior to that conducted on a 3D model simulator in terms of organizational flexibility and ductility and tactility of soft tissue during suturing (P = 0.04 and P < 0.01, respectively). Similarly, a histological study of a porcine tongue revealed that the muscular component occupies approximately 80% of the total of the tongue’s total tissue [18]. Therefore, since muscle reconstruction remains an incremental process in cleft palate repair, the large muscular component of the porcine tongue would favor such simulators over 3D printed simulators. The tip of the porcine tongue seemed to be the appropriate size for learners to practice the surgical procedure and actually understand the fundamental surgical principle. Unlike the 3D-printed simulator, surgical procedures on animal-derived simulators, such as incision, tissue dissection, suturing and tissue handling, in a controlled and repeatable environment, may be more similar to those performed on human tissues, this makes them an excellent choice for practicing palatoplasty procedures, which involve delicate manipulation of soft tissues [18]. Also, Porcine tongue simulators are relatively accessible and cost-effective compared to simulators, making them a practical choice for surgical education programs with budget constraints. On the other hand, the previous study had no evaluation of surgical skills following the module, where the readiness of the lecture for technical help facilitated the participants’ good performance. The size of the porcine tongue and surrounding structures need specific modifications, which may influence practice. In addition, the porcine tongue can be fixed with support brackets that are not yet commercially available [18].

In the present study, there were no significant differences between the simulators in terms of anatomical design simulation and incision simulation. Hence, the potential of porcine tongue and 3D-printed models in simulating the surgical procedure of double-opposing Z-plasty of palatoplasty are similar. Huang et al. [18] reported that the porcine tongue-based cleft palate simulator can help residents to practice double-opposing Z-plasty of palatoplasty. Cote et al. [19] developed a 3D-printed haptic simulator for von-Langenbeck palatoplasty simulation and reported that the 3D-printed cleft palate simulator performed well in terms of anatomical correctness, tissue similarity, and the ability to carry out all the steps of von-Langenbeck palatoplasty. There has been an increase in the use of 3D-printed simulators in surgical training because of their enhanced anatomical visualization [25]. The 3D-printed cleft palate simulator introduced by Simulate Medical Corporation can provide a high-fidelity replica of the anatomy of the cleft palate and make surgical training for palatoplasty more realistic [26]. Ahmed et al. [16] concluded that 3D-printed simulators are an excellent teaching tool with the added advantage that medical institutions may utilize them to develop accurate anatomical simulation collections that include specific case variations.

The results of our questionnaire demonstrated that 94.4% of participants favored the 3D-printed simulator if it was well improved to simulate the palatal muscles and tissues. Concurrently, a study evaluating the use of 3D-printed models of cleft lip and palate as an educational tool reported that most students believed that having a 3D-printed model on hand would aid them in self-study [16].

Overall, both porcine tongue and 3D-printed simulators have advantages and disadvantages for simulating palatoplasty. Unlike the porcine tongue simulator, the 3D-printed simulator mimics the face and oral cavity, which is crucial for surgical training. Surgeons can gain a better understanding of the spatial relationships and structures they’ll encounter during palatoplasty [25, 27–29]. 3D-printed simulators provide a customizable simulation of the patient’s anatomy and defect geometry, allowing educators to design and create specific surgical scenarios tailored to the needs of trainees; in particular, understanding the defect extension and anatomical variation may be more suitable for preoperative planning and optimization of surgical procedures [30, 31]. Furthermore, a self-studying aid that has a major role in motivation with anatomic visualization, a crucial element in the learning process. Moreover, trainees can repeat procedures on 3D-printed simulators as many times as needed to build muscle memory and confidence [16]. This repeatability helps refine surgical techniques and reduce the learning curve. Additionally, a 3D-printed simulator allows for an infinite number of types of clefts with simulated faces and oral cavities, and several repair techniques might be tried on the models in the future [19, 29]. These simulators eliminate the potential risks associated with practicing on live animals or cadavers, such as cross-infection.

It’s important to acknowledge the study limitations to interpret the results accurately and guide future studies. First, the small sample size. Second, the study focused on evaluating the simulators for Furlow double-opposing Z-plasty. Different cleft palate repair techniques exist, and the findings may not be applicable to other techniques. Third, the evaluation of simulator performance relied on subjective evaluations by senior cleft surgeons. Therefore, a large sample size involving resident and senior cleft surgeons’ study is still recommended to confirm the present findings.

## Conclusion

Simulation-based training plays a critical role in expanding the focus on competency-based training. Both porcine tongue simulator and 3D-printed simulator have their unique strengths in surgical education for palatoplasty. Thus, the combined use of both cleft palate simulators is efficient as an educational model to practice Furlow double-opposing Z- palatoplasty with superiority of porcine tongue simulator in terms of organizational flexibility and ductility and suturing simulators. With the 3D-printed simulator, various palatoplasty techniques can be repeatedly practiced with better-simulated face and oral cavity.

## Data Availability

The datasets used and/or analysed during the study are available from the corresponding author on reasonable request.
