# Correction: Two cleft palate simulators of Furlow double-opposing Z- palatoplasty: a comparative study

**DOI:** 10.1186/s12893-023-02276-0

**Published:** 2024-01-02

**Authors:** Sadam Ahmed Elayah, Mohammed Qasem Al-Watary, Karim Ahmed Sakran, Yang Chao, Li Jingtao, Huang Hanyao, Yang Li, Bing Shi

**Affiliations:** 1 State Key Laboratory of Oral Diseases & National Center for Stomatology & National Clinical Research Center for Oral Diseases, West China Hospital of Stomatology, Sichuan University, Chengdu, 610041 China; 2 Department of Oral and Maxillofacial Surgery, Cleft Lip and Palate Center, Faculty of Dentistry, Jiblah University for Medical and Health Sciences, Ibb, Yemen

**Correction: BMC Surgery (2023) 23:1**

**https://doi.org/10.1186/s12893-023-02201-5**

Following publication of the original article [1], the wrong figure appeared as Fig. 1.; the figure should have appeared as shown below.

**Fig. 1:**
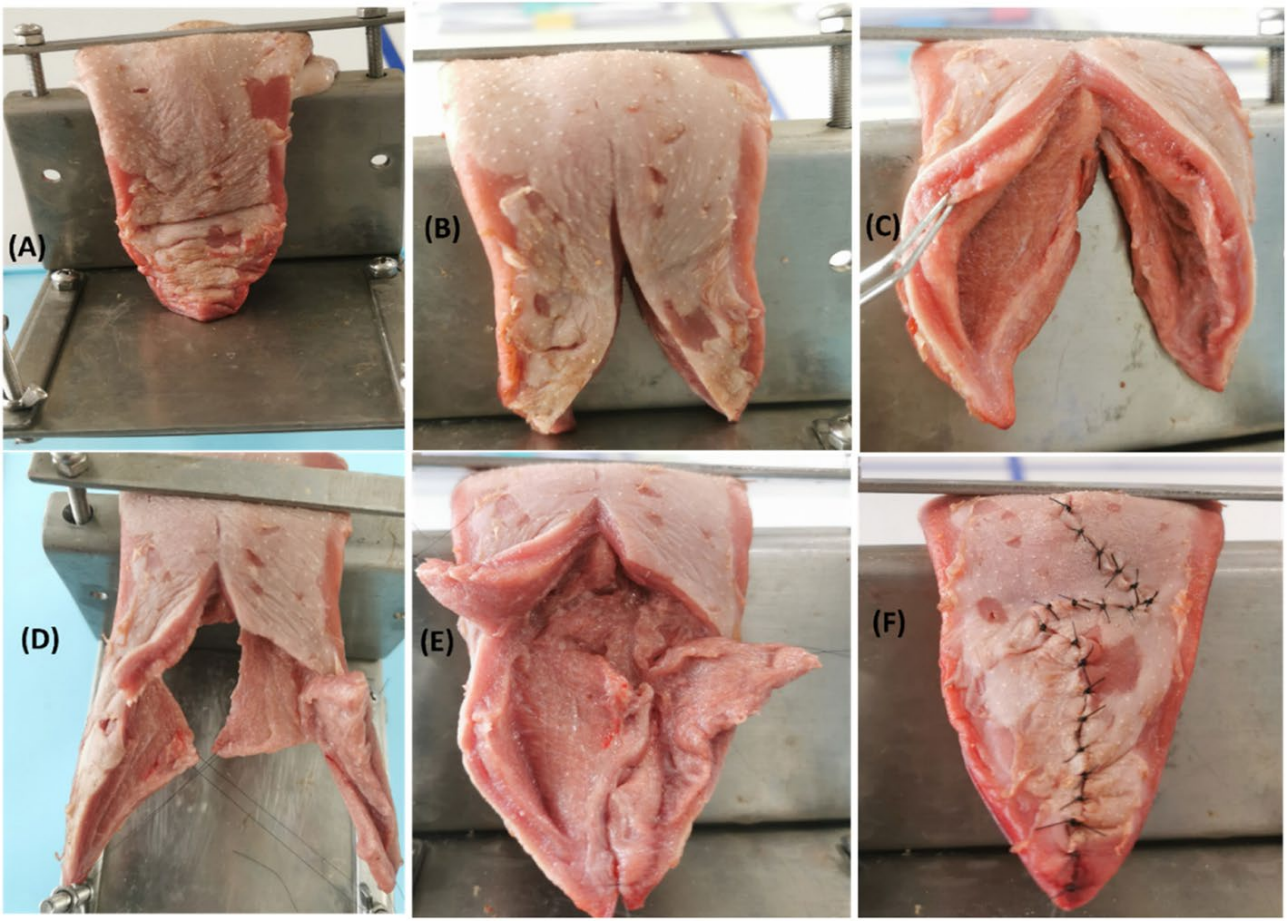
The surgical procedure of double-opposing Z-plasty on porcine tongue; (**A** & **B**) The porcine tongue is fixated on the holder with being cut in the middle part. (**C**) To simulate the Nasal and oral layers separation. (**D**) To simulate the Z-plasty flaps, Preparation of two myomucosal flaps and two mucosal flaps. (**E**) Suturing of Nasal Z-plasty flap. (**F**) Suturing of Oral Z-plasty flap

The original article has been corrected.
